# Nutrigenomics in Regulating the Expression of Genes Related to Type 2 Diabetes Mellitus

**DOI:** 10.3389/fphys.2021.699220

**Published:** 2021-07-21

**Authors:** Karoline Felisbino, Juliano Gomes Granzotti, Larissa Bello-Santos, Izonete Cristina Guiloski

**Affiliations:** ^1^Centro de Ensino Superior de Maringá (CESUMAR), Curitiba, Brazil; ^2^Programa de Pós-graduação em Biotecnologia Aplicada à Saúde da Criança e do Adolescente, Faculdades Pequeno Príncipe, Curitiba, Brazil; ^3^Instituto de Pesquisas Pelé Pequeno Príncipe, Curitiba, Brazil

**Keywords:** nutrigenomics, type 2 diabetes mellitus, chronic disease, bioactive compounds, nutrients, gene-nutrient interactions, polyphenols

## Abstract

Nutrigenomics is the study of the gene-nutrient interaction and it indicates that some nutrients, called bioactive compounds, can mold the genetic expression or change the nucleotide chain. Polyphenols are secondary metabolites found in plants that are regularly consumed in functional foods and help prevent or delay the onset of type 2 diabetes mellitus (T2DM) and its complications. This article objected to review studies about the interaction of diet with polyphenols and Mediterranean diet in the expression of human genes related to T2DM. Resveratrol acts as an antioxidant, anti-inflammatory, and increases mitochondrial function. Regular consumption of quercetin resulted in improvement of hypertension and suppression of diabetes-induced vasoconstriction. Genistein also showed positive results in T2DM, such as increased cell mass and improved glucose tolerance and insulin levels. Catechins showed efficiency in inducing genes in triacylglycerol biosynthesis, inhibition of fatty acids and cholesterol, and resulting in their participation in mitigating complications of diabetes. Lastly, curcumin was demonstrated to be a protector of the pancreatic islets against streptozotocin-induced oxidative stress. Growing evidence suggest that bioactive compounds such as polyphenols have an important role in T2DM and the prevention and treatment of its complication, as they cause activation or inhibition of related genes.

## Introduction

Diabetes mellitus (DM) is a syndrome of multiple etiologies, characterized mainly by chronic hyperglycemia with dysfunctions related to the metabolism of proteins and lipids. The increase in glucose concentrations in the bloodstream (hyperglycemia) may be associated with the inability to produce, secrete, or fail to absorb insulin, or even with a set of all these abnormalities (Kuzuya et al., 2002).

Among the types of diabetes, type 2 diabetes mellitus (T2DM) represents 90% of cases, and it occurs when the body does not properly use the insulin produced or does not produce the required hormone capable of controlling blood glucose (Holman et al., 2015). Some of the patients do not present, at the beginning of metabolic alterations, symptoms, such as thirst, increased diuresis, leg pains, and visual alterations, however, these can manifest late, becoming aggravating factors, and when the diagnosis is not made early, the complications generated by the disease can be greater. The treatment usually consists of changes in eating habits, physical exercise, and pharmacological therapy (Zheng et al., 2018).

According to Steemburgo et al. (2009), several chronic diseases, such as T2DM, have their pathogenesis associated with genetic and environmental aspects. Among the latter, the diet has the power to contribute to the incidence and the severity of these pathologies. Nevertheless, the diet can have a modulating action on phenotypes linked to genetic changes, and this action is related to gene and nutrient interaction.

A variety of habits and environmental factors, including food, can influence the expression of genes involved in T2DM which could be beneficial or harmful in relation to disease. Great progress has been made in the study of these interactions after the Human Genome Project and with the emergence of genetic nutrition, a field of nutrition that studies the relationship between genome and eating habits (Billings and Florez, 2010; Cole and Florez, 2020).

In this perspective, genetic nutrition highlights what is most recent in the science of nutrition. The concepts of nutrigenomics and nutrigenetics are related but follow a different approach to the understanding of the association between genes and diet. Nutrigenomics studies the nutrients and food structures capable of acting on the expression of genes, in contrast, nutrigenetics studies the variables of the personal genome in relation to how we respond to foods or compounds consumed in a diet (Mickelson et al., 2019). Polyphenols are among these compounds. They are secondary metabolites produced by plants that are part of the human diet. They have the potential to interact with genetic material and may alter the expression of important genes. In addition, they act as antioxidants, anti-inflammatories have been studied in the prevention and treatment of type 2 diabetes (Nunes et al., 2018; Li et al., 2019).

Thus, this study reviewed the relationship between polyphenols and gene expression in T2DM identifying major genes and scientific evidence.

## Diabetes Mellitus

Diabetes mellitus is a metabolic disorder characterized by persistent hyperglycemia in the bloodstream as a result of the disabled action and/or failure in production of the hormone insulin, which has as function to promote glucose entry into cells (Kuzuya et al., 2002; Holman et al., 2015). When insulin is absent or its function is impaired, cells are unable to absorb glucose, which remains in the bloodstream causing hyperglycemia (Asmat et al., 2016).

There are three main types of diabetes: type 1 diabetes mellitus, type 2 diabetes, and gestational diabetes. T2DM stands out among them by being present in about 90% of cases (Cole and Florez, 2020). T2DM is a multifactorial polygenic disease, which is believed to be a result of interaction between multiple genes and environmental factors (Rheinheimer et al., 2017).

According to Mahler and Adler (1999), the pathophysiology of T2DM includes peripheral resistance to insulin, increased hepatic glucose production, and functional impairment of pancreatic cells. In the initial stage of the disease, a decrease in insulin sensitivity known as insulin resistance is observed and, to compensate, pancreatic cells increase insulin secretion resulting in a state of hyperinsulinemia. As the disease progresses, these cells lose the ability to secrete large amounts of insulin to maintain balance and the individual develops a deficiency of this hormone (Asmat et al., 2016; American Diabetes Association, 2020). The main characteristic of T2DM is the development and persistence of hyperglycemia, which occurs in conjunction with hyperglucagonemia and increased hepatic glucose production (García-Chapa et al., 2017; Furmli et al., 2018). Multiple metabolic disorders, such as impaired lipid and lipoprotein metabolism, oxidative stress, subclinical inflammation, vascular endothelial dysfunction, and hypertension accompany T2DM (Spranger et al., 2003; Gadi and Samaha, 2007). These disorders have long-term consequences, such as micro and macrovascular complications, neuropathy, retinopathy, nephropathy, and therefore increased mortality rate (Lloyd et al., 2001; Constantino et al., 2013).

## Oxidative Stress and Diabetes

Free radicals are highly reactive molecules that contain oxygen (or nitrogen) and are naturally generated in small amounts during metabolic reactions. Oxidative stress is an imbalance that occurs when the production of free radicals exceeds the antioxidant defenses resulting in damage to vital biomolecules to membranes and DNA, proteins, and lipids (Wu and Cederbaum, 2003).

The cellular damage caused by these reactive oxygen species (ROS) is related to the pathological process of diseases such as cancer and T2DM (Dandona et al., 1996). Hyperglycemia in DM induces an increase in oxidative stress, favoring the progression, and the appearance of complications of the disease (Nishikawa et al., 2000; Rajendran et al., 2011). The reduction of oxidative stress can happen due to antioxidants, molecules that play an important role against free radicals, acting in order to eliminate them or transform them into less toxic products for the cell (Sies, 1993).

Insulin resistance and pancreatic beta-cell dysfunction are associated with oxidative stress. Diabetic patients showed lower enzyme and antioxidants levels, low markers of oxidative stress, and increased production of ROS, which can contribute to vascular complications in DM (Dandona et al., 1996; Weyer et al., 1999; Kaneto et al., 2007; Jiménez-Osorio et al., 2014).

## Nutrigenomics and Dietary Factors

Nutrigenomics studies how nutrients affect gene expression (Marcum, 2020), bringing the perspective of designing and prescribe customized diets according to the individual genetic makeup and expanding strategies for prevention and treatment of non-communicable diseases (NCDs), such as obesity, T2DM, inflammatory bowel disease (IBD), and cancer (Fialho et al., 2008). It seeks to observe the variations of genetic polymorphisms, responsible for the absorption, metabolism, and excretion of nutrients and bioactive compounds, acting in conjunction with other sub-areas of studies, including metabolomics, transcriptomics, and proteomics (Dimitrov et al., 2016) that together, allow the discovery of the influences of nutrients in the epigenome or genome and how each individual can be affected (Rist et al., 2006).

The diet alone or in conjunction with other environmental factors may cause epigenetic changes (Fenech et al., 2011), and these changes in the genes have great influences on cellular processes associated with health and disease, hormonal balance, cell signaling, carcinogen metabolism, apoptosis, cell cycle control, changes in energy levels, and angiogenesis (Ferguson, 2006). In addition, offspring can also be affected through embryonic development and long-term health (Trujillo et al., 2006). Therefore, it is necessary to understand the health status and correlate it with the individual nutritional needs (Picó et al., 2019).

Functional foods are then able to interact with the genome, being defined as foods that contain physiologically active components that perform a beneficial function to health in addition to the basic nutritional function (Henry, 2010). These components are called bioactive compounds that, even when present in small amounts, their frequent intake has the ability to reduce the risk of chronic diseases. It is recommended that these compounds can be obtained in their natural form. As examples of bioactive compounds, we can mention the polyphenols, such as resveratrol, quercetin, curcumin, and genistein (Karasawa and Mohan, 2018).

The genes may change during intrauterine life, when the nutrients and other food compounds can modulate gene expressions or even change the nucleotide sequence and modify the response of the organism in the presence of toxic and infectious compounds, in addition, the inherited individual genetic sequence can also influence diet, leading to the suppression of nutrients and risks for NCDs. The knowledge of these interactions between the genome and food contributes to the promotion of health and reduces the risks for NCDs through personalized diets (Paparo et al., 2014; Raiten and Bremer, 2020).

## Polyphenols and Diabetes Genes

The Mediterranean diet, rich in polyphenol and others nutrients, consists of a balanced intake of fruits, vegetables, fish, cereals, and polyunsaturated fats, combined with a reduction in the consumption of meat and dairy products and a moderate intake of alcohol, mainly red wine (Di Daniele et al., 2017). This diet has been working to prevent different metabolic disorders such as cardiovascular disease and T2DM, and has been shown to decrease the incidence of neurodegenerative diseases and cancer (Bach-Faig et al., 2011). The application of the Mediterranean diet resulted in a reduction of the rate of diabetes incidence by 52% (Salas-Salvadó et al., 2011).

**Figure 1:**
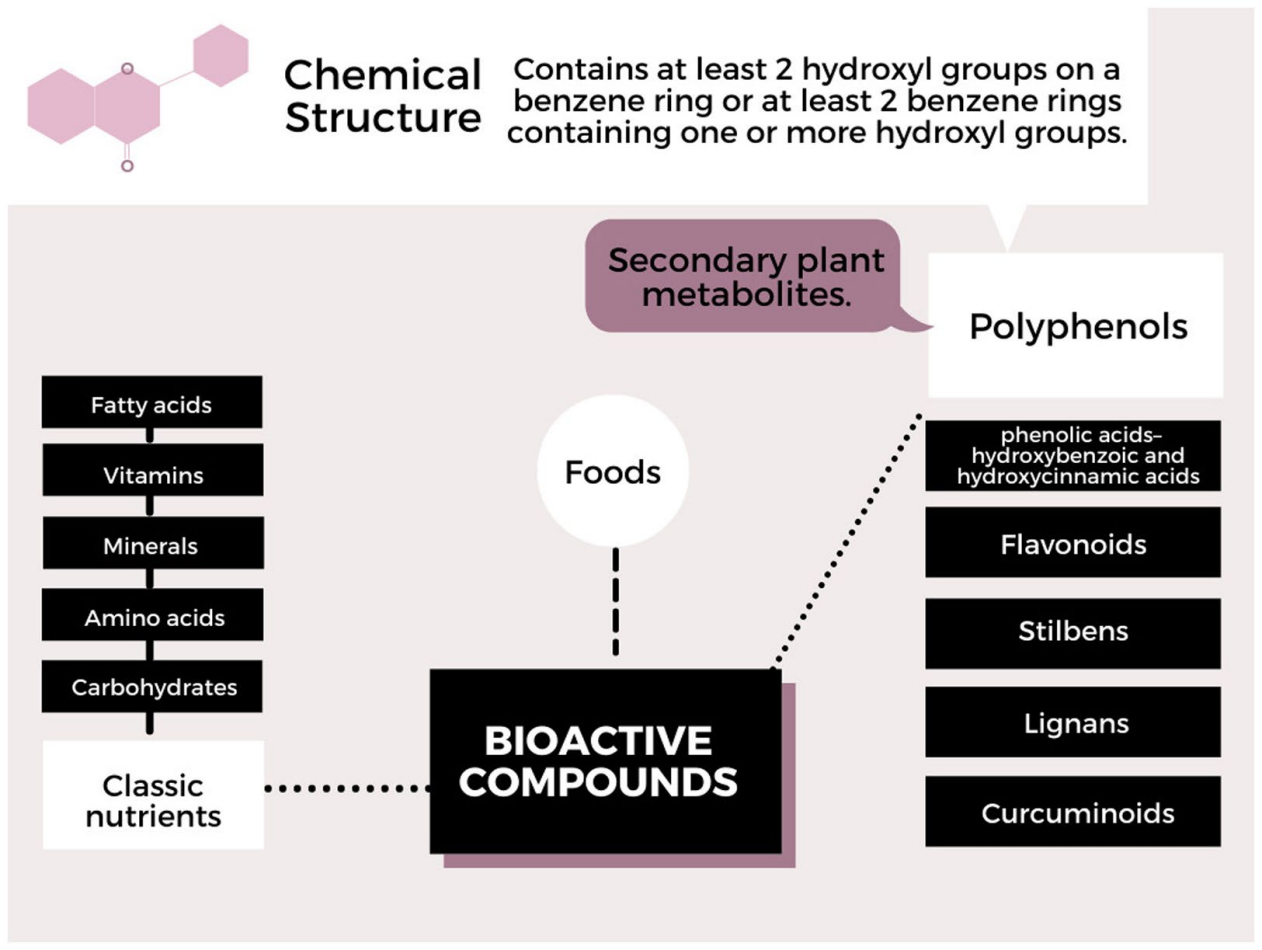
Plant phenolics classes categorized according to their chemical structures.

Polyphenols can interact with the DNA molecule, RNA, or with proteins involved in the activation cascade, changing number, function, and structure. Generally epigenetic mechanisms, such as methylation, DNA demethylation, and histone modifications, whether by phosphorylation, acetylation, or others, may arise from the interaction between the compounds found in food and the genes involved. These modifications are reproduced in the phenotype that can change the state of health and disease. But it is still very complex, due to genetic variability, interaction complexity, and variation in the mode of action of polyphenols. The classification of polyphenols according to the chemical structure is shown in Figure 1 (Nunes et al., 2018; Papuc et al., 2020). Furthermore, the absorption and metabolism of polyphenols in the human body (stomach, intestine, and liver) may have a different impact on human health, and factors, such as bioavailability, intestinal microbiota, and transport proteins, and the type of polyphenol may affect the bioactivity of the consumed polyphenol (Scalbert et al., 2002; Manach et al., 2004; Hoda et al., 2019).

Polyphenols can interact with the epigenome in different ways, which can alter gene expression, causing inhibition or activation. Curcumin, for example, can cause demethylation and interact with transcription factors; catechins such as epigallocatechin-3 gallate (EGCG) can also reduce the methylation mechanism by inhibiting the DNA methyltransferase enzyme and cause phosphorylation of serine and tyrosine residues of histone proteins. Flavonoids, such as Luteolin and genistein can cause acetylation of histone H3 and cause hypermethylation of genes and cause inhibition, as well as resveratrol and folic acid (Han, 2003; Collins et al., 2007; Berner et al., 2011; Vetterli et al., 2011; Goh et al., 2014; Boyanapalli and Kong, 2015; Li et al., 2015).

## Genes Related to Type 2 Diabetes Mellitus

Oxidative stress is associated with T2DM (Jiménez-Osorio et al., 2014), therefore, genes as *NFE2* and *NFE2L2* with a regulatory role in the expression of antioxidant proteins can be targeted for protection against oxidative stress (Fu et al., 2017). Animal models showed that Nrf2 agonists improved insulin resistance and obesity, and prevented pancreatic beta-cell apoptosis (Zhao et al., 2011; Bhakkiyalakshmi et al., 2014; Matzinger et al., 2018).

The *PRKAA2* gene (AMPK) is responsible for preventing the production of glucose, cholesterol, and triglycerides by promoting the oxidation of fatty acids. This gene has a relationship with the *SIRT1* gene because its functioning results in the activation of the *SIRT1* gene, and this causes an increase in the substrate NAD+. The *SIRT1* gene is responsible for deacetylation processes and modulation of several other genes and therefore can control hepatic glucose production, lipid metabolism, and sensitivity and insulin production. It may, for example, regulate the activity of peroxisome proliferator-activated receptor gamma coactivator 1 alpha (PGC-1) causing its deacetylation, which has the function to suppress the production of ROS and regulate mitochondrial biogenesis. And lastly, it is able to reduce the production of hepatic glucose by deacetylation and activation of adenosine monophosphate-activated protein kinase (AMPK; Price et al., 2012; Rato et al., 2014). Therefore, there is a reciprocal activation between *AMPK* and *SIRT1*, which is suspended by hyperglycemia, decreasing the expression of *AMPK* and consequently decreasing the expression of *SIRT1* (Cantó et al., 2009; Clarke et al., 2014; Li et al., 2019).

Other genes are related to insulin signaling, activation, and production, such as the *PI3KR1*, *IRS1*, *FFAR1*, *HNF4A*, and *ENPP1* genes. The *PI3KR1* gene encodes a phosphoinositide-3-kinase regulatory subunit 1 enzyme with direct function in the insulin signaling pathway (Karadoğan et al., 2018). The *IRS1* gene encodes the insulin receptor substrate-1, which after phosphorylate regulates growth cascades, metabolism, and glucose transporter (Keshavarzi and Golsheh, 2019). The *FFAR1* gene carries the code for the formation of the Free fatty acid receptor 1 protein (Ffar1). This protein and agonists (substances capable of activating Ffar1) can amplify insulin secretion in pancreatic beta cells and control blood glucose (Kohara et al., 2019). The *HNF4A* gene acts by maintaining glucose homeostasis (Azizi et al., 2019) as it directly activates the expression of the insulin gene. In addition, SNPs in the promoter region of the *HNF4A* gene were correlated with predisposition to T2DM (Bartoov-Shifman et al., 2002). Finally, the *ENPP1* gene inhibits insulin receptor signaling, which is why it is related to the development of insulin resistance when overexpressed (Bacci et al., 2007; Neamati et al., 2017).

Some genes are more directly related to pancreatic β cells. The *IGF2BP2* gene plays an important role in regulating the function of pancreatic cells (Huang et al., 2010) and its deregulation is associated with insulin resistance (Cao et al., 2018). In studies with rats, total ablation of *IGF2BP2* results in increased insulin sensitivity and glucose tolerance (Yang et al., 2020). The overexpression of the *PARP1* gene is associated with tissue damage and destruction of β cells, being a highly relevant factor in endothelial dysfunction in diabetes (Garcia Soriano et al., 2001; Pacher and Szabo, 2005).

Glucose transport and production also stand out as important processes in T2DM. The genes *SLC2A1* and *SLC2A2*, for example, encode proteins that transport glucose into the cells, which reduces blood sugar and prevents disease (Kilpeläinen et al., 2007; Fu et al., 2017). The *PCK1* and *PCK2* genes encode proteins related to the production of glucose and have increased expression in people with diabetes (Cao et al., 2004). The reduction in the expression of the *TCF7L2* gene was associated with an increase in β cell apoptosis (Shu et al., 2008) and an increase in hepatic glucose production and a reduction in insulin secretion (Lyssenko et al., 2007). The overexpression of the *G6PC* gene was observed in glucose intolerance and hyperinsulinemia (Im et al., 2011). Estrogens can also regulate the transport and control the levels of glucose in the adipose tissue and muscle, for example, so the gene *ESR1* can also be targeted in the treatment or prevention of T2DM (Barreto-Andrade et al., 2018).

Finally, we can also mention genes related to inflammatory processes and oxidative stress. Hyperglycemia promotes the formation of advanced glycation end-products (AGEs) that induce inflammation and oxidative stress, so polymorphisms in the *AGER* gene have been associated with the risk of type 2 diabetes (Kang et al., 2012; Lin et al., 2012). In addition, the inhibition of the NFKB1 and NFKB2 genes shows a decrease in the inflammatory process, consequently improving hypertension and suppressing vasoconstriction induced by diabetes (Gautam et al., 2017; Behera et al., 2020). In patients with T2DM, we can also observe an increased expression of the *FTO* gene, which may be involved in oxidative metabolism, lipogenesis, and oxidative stress (Bravard et al., 2011).

The functions associated with T2DM of the commented genes, in addition to the respective bioactive compounds found in foods that show some type of interaction are described in Table 1.

**Table 1:** Genes associated with type 2 diabetes mellitus (T2DM) and respective bioactive compounds found in foods that are capable to change in expression.

Genes	Protein	Function	Related foods	References
*NFE2L2/NRF2*	Nuclear factor erythroid 2 like 2	Regulator of the expression of antioxidant proteins.	Animals and human cells showed that curcumin is a substance capable of reducing oxidative stress in different target (cardiac, muscle, hepatic, etc.) for mechanism epigenetic, more specifically demethylation, that activating *NFE2L2/NRF2* gene and can be used in the prevention and treatment of diabetes.	Zhao et al., 2011 He, 2012 Bhakkiyalakshmi et al., 2014 Byun and Lee, 2015 Houghton et al., 2016 Matzinger et al., 2018 Parsamanesh et al., 2018
*NFE2*	Nuclear factor, Erythroid 2	Regulator of the expression of antioxidant proteins.	Studies with humans and animals show the consumption of EGCG and curcumin is related to the increase in NFE2. These are substances that can reduce the methylation mechanism of the gene, that is, it acts as an epigenetic compound, capable of activating the gene. EGCG green tea inhibits DNA methyltransferase, as does curcumin, which is also capable of regulating histone changes.	Boyanapalli and Kong, 2015 Chen et al., 2015 Wang et al., 2015 Fu et al., 2017
*PRKAA2*	5’-AMP-activated protein kinase catalytic subunit alpha-2	It encodes the protein kinase that is activated by AMP molecules. The general function of this protein is to turn on important metabolic pathways for energy production and turn off the pathways that spend a lot of ATP, controlling the body’s need for energy according to the situation.	Studies with rats show the quercetin is a flavonoid present in a variety of foods, such as red onion, broccoli, and apple, and has anti-inflammatory, antioxidant, and anti-apoptotic properties. This flavonoid acts on glucose homeostasis in skeletal muscle, increasing glucose uptake by stimulating GLUT4 translocation by activating adenosine monophosphate-activated protein kinase (AMPK) and in the liver, also by activating AMPK where it resulted in the suppression of glucose-6-phosphatase reducing hepatic glucose production.	Nijveldt et al., 2001 Cantó et al., 2009 Price et al., 2012 Clarke et al., 2014 Eid et al., 2015 Li et al., 2019
*SIRT1*	NAD-dependent protein deacetylase sirtuin-1	It encodes the protein called Sirtuin 1 that belongs to the family of proteins that interact with the genetic material causing deacetylation of histones, that is, it is able to inactivate genes by an epigenetic mechanism.	Resveratrol has allosteric effect and is an excellent activator of the SIRT gene and has been used as a treatment for diabetes by normalizing hyperglycemia, improving insulin sensitivity, decreasing liver glucose production, and regulating mitochondrial biogenesis and lipid metabolism. However, we find a complexity in relation to the mode of action and effect of this polyphenol and dependent on concentration. Studies with human muscle cells have shown that at high concentrations it can be harmful and inhibit mitochondrial respiration.	Howitz et al., 2003 Cantó et al., 2009 Vetterli et al., 2011 Price et al., 2012 Clarke et al., 2014 Goh et al., 2014 Rato et al., 2014 Williams et al., 2014; Yacoub et al., 2014 Tavakoli Faradonbeh et al., 2020
*PI3KR1*	Phosphoinositide-3-kinase regulatory subunit 1	It forms a protein involved in insulin signaling, cancer, and cytokines (involved in the immune system), and also in adipocyte maturation.	There are few scientific articles related to nutrigenomics and the *PI3KR1* gene.	Hale et al., 2012 Thauvin-Robinet et al., 2013 Gao et al., 2014 Karadoğan et al., 2018
*IRS1*	Insulin receptor substrate 1	Insulin signaling.	EGCG at low concentrations does not demonstrate activation of the IRS-1 gene, but it does show to be an inhibitor of gluconeogenesis in isolated hepatocytes. However, polyphenol-rich green tea increased the expression of the IRS1 gene in the muscle of the rat. The polyphenol-rich ethyl acetate fraction isolated from *Molineria latifolia* improves insulin resistance in experimental diabetic rats through the activation of IRS1/AKT, by altering the phosphorylation of gene-related serine and tyrosine residues.	Collins et al., 2007 Qi et al., 2011 Yang et al., 2017 Ooi et al., 2018 Keshavarzi and Golsheh, 2019
*FFAR1*	Free fatty acid receptor 1	Metabolic regulation of insulin secretion and hepatic glucose uptake *in vitro*.	Humans cells demonstrated that the anthocyanins present in purple corn have the possibility of activating the *FFAR1* gene, a known marker that, when activated, can contribute to the treatment of type 2 diabetes and its complications. Another article also shows that some polyphenols, such as anthocyanin, can activate the FFAR1 gene in pancreatic Beta cells, and point to antidiabetic potentials for prevention and treatment.	Wagner et al., 2014 Luna-Vital and De Mejia, 2018 Papuc et al., 2020
*HNF4A*	Hepatocyte nuclear factor 4 alpha	Regulator of hepatic gluconeogenesis and insulin secretion.	Luteolin, a flavone present in chamomile, peppers, and celery tea, has a lipid-lowering effect by suppressing *HNF4A* gene in mouse cells, by epigenetic means, related to histone H3 acetylation.	Stoffel and Duncan, 1997 Wang et al., 2000 Silander et al., 2004 Li et al., 2015
*ENPP1*	Ectonucleotide pyrophosphatase/phosphodiesterase 1	Transmembrane glycoprotein with effect on insulin signaling and glucose metabolism.	There are no articles related to foods or bioactive compounds in the modulation or control of the expression of the *ENPP1* gene. However, it has been observed that zinc deficiency can impair the activities of some ectoenzymes, including *ENPP1*.	Hale et al., 2012 Neamati et al., 2017 Gohari-Lasaki et al., 2020
*IGF2BP2*	Insulin-like growth factor 2 mRNA-binding protein 2	Regulator of cellular metabolism.	Diet with less protein intake showed a relationship with the increase in *IGF2BP2*. But no studies on polyphenols or Mediterranean diet and changes in the expression of this gene have been reported.	Rao et al., 2016 Gokarn et al., 2018 Hu et al., 2020
*PARP1*	Poly(ADP-Ribose) polymerase 1	DNA damage signaling.	It is possible to observe the protection of curcumin in pancreatic islet cells exposed to streptozotocin, the compound can decrease the formation of reactive oxygen species (ROS) and inhibit the activation of the poly ADP-ribose polymerase-1 enzyme, encoded by the *PARP1* gene, and can also prevent the reduction of ROS levels of free radical scavenging enzymes. In HUVECs cells, it was observed that flavonoids (rutin, quercetin, and flavone) can inhibit *PARP* activation and improve diabetes complications. These compounds can interact with transcription factors and regulate gene expression.	Pacher and Szabo, 2005 Meghana et al., 2007 Tunon et al., 2009 Boesten et al., 2015 Narne et al., 2016
*SLC2A1* *SLC2A4*	Solute carrier family 2, facilitated glucose transporter member 1, and solute carrier family 2 member 4	They encode glucose transporters (GLUT 1 and GLUT 4).	A variety of polyphenols, such as catechins, flavonoids, phenolic acids, and among others, are related to the increase in glucose transporters in animals and human cells.	Hanhineva et al., 2010 Wang et al., 2015 Fu et al., 2017
*PCK1* *PCK2*	Phosphoenolpyruvate carboxykinase 1	The *PCK1* gene carries the code for the formation of an enzyme called phosphoenolpyruvate caboxycin (PEPCK). This enzyme is a limiter of gluconeogenesis, that is, it regulates the speed of this process.	The plant *Juniperus procera*, rich in polyphenols, was able to reduce the expression of the *PEPCK* gene in diabetic rats, in liver and kidney cells, serving as a treatment for hyperglycemia, with anti-inflammatory and hypoglycemic effects.	Cao et al., 2004 Alkhedaide et al., 2019
*TCF7L2*	Transcription factor 7-like 2	Wnt signaling (β cell proliferation and secretion of the insulin).	The gene is expressed in adipose tissue and SNPs are already being associated with diabetes risk. A randomized clinical trial with people at high cardiovascular risk shows that Mediterranean diet can reduce the adverse effect of the rs7903146 (TT) polymorphism and reduce fasting blood glucose and lipids, in addition to preventing stroke.	Corella et al., 2013 Haddad et al., 2017 Beloso et al., 2018 Grant, 2019
*G6PC*	Glucose-6-phosphatase catalytic subunit	Liver glucose production during fasting or T2DM.	Ingestion of EGCG is also related to the control of gluconeogenesis by suppressing the expression of the glucose-6-phosphatase gene. Treatment with the extract of the saffron stigma reduced the expression of the *G6PC* gene in diabetic rats. Studies with quercetin demonstrated that this compound activated AMPK and resulted in the suppression of the gene, decreasing the production of hepatic glucose since AMPK negatively regulates *G6PC*.	Han, 2003 Im et al., 2011 Eid et al., 2015 Al-Daghri et al., 2017 Motamedrad et al., 2019
*ESR1*	Estrogen Receptor 1	It encodes a transcription factor that responds to the action of estrogen and cancer.	In a study with CACO-2 cells, high-concentration folic acid has been shown to cause methylation in the ESR1 gene; zebularine decreased the methylation of the gene; genistein caused hypermethylation of the *ESR1* gene promoter; resveratrol increased the expression of *ESR1* and EGCG induced *ESR1* hypermethylation and a non-significant decrease in the expression of the *ESR1* gene.	Wang et al., 2006 Berner et al., 2011 Hale et al., 2012
*AGER*	Advanced glycosylation end-product specific receptor	Specific recipient of advanced glycation end products.	Curcumin may have antioxidant and anti-inflammatory properties, and attenuate oxidative stress induced by AGEs, suppressing the expression of the *AGER* gene in mouse liver cells and cardiac tissue. It has the ability to interact with transcription factors such as NFκB, which reduces gene transcription.	Kang et al., 2012 Lin et al., 2012 Abdel-Mageid et al., 2018 Egaña-Gorroño et al., 2020
*NFKB1* *NFKB2*	Nuclear factor kappa B subunit 1, Nuclear factor kappa B subunit 2	Transcription factor involved in anti-inflammatory pathways.	Flavonoids (fisetin, apigenin, quercitin, chrysin, isoliquiritigenin, rutin, genistein, and others) have anti-inflammatory, antioxidant, and anti-apoptotic properties. These biocompounds can cause inhibition of NF-κB, mostly by reducing phosphorylation of proteins, and thus can improve vascularization in diabetics and reduce the risk of hypertension, which has already been observed in different tissues of humans and animals.	Tribolo et al., 2008 Mahmoud et al., 2013 Gautam et al., 2017 Choy et al., 2019 Behera et al., 2020
*FTO*	Alpha-ketoglutarate-dependent dioxygenase	It forms a nuclear protein involved in insulin signaling, ROS production, and adipose tissue development.	Studies in humans and animals have shown that the Mediterranean diet has an influence on the *FTO* gene and that it is related to the development of type 2 diabetes. There is a suggestion that the diet can neutralize the genetic predisposition related to the *FTO* gene, but nothing very explanatory.	Bravard et al., 2011 Ortega-Azorín et al., 2012 Loos and Yeo, 2014 Yang et al., 2017 Di Renzo et al., 2018

*EGCG, epigallocatechin-3 gallate; CACO-2 cells, human colon carcinoma cell line; and HUVECs cells, human umbilical vein endothelial cells*.

## Future Perspectives

One way to prevent T2DM is to know the related genes and define the foods that can interact with them in a positive way. The results demonstrate that the active ingredients found in some foods, such as resveratrol, quercetin, genistein, catechins, curcumin, and anthocyanins, interact with DNA and show protective effects in relation to T2DM. These compounds interact with related genes mainly in the control of insulin secretion and signaling, oxidative stress, inflammatory processes, cellular apoptosis, and glucose and lipid metabolism. With that, we can say that the bioactive compounds present in functional foods have established functions in the prevention and treatment of T2DM and its complications. Many of the described genes need further studies to become more important in the prevention and treatment of T2DM and other diseases, like the *IRS1*, *TCF7L2*, *IGF2BP2*, *PI3KR1*, *PCK1*, *PCK2*, and *FTO* genes that are related to the etiology or control of the disease but there are not many studies on compounds that can modulate their expression. The *PRKAA* and *SIRT1* genes, on the other hand, are very well-studied and, therefore, their manipulation could be used to benefit patients with T2DM or prevent the disease, since both genes, besides being associated with each other, interfere in the expression of other genes. Most studies only seek to know whether there is a change in the expression level of genes or not, there is a lack of information about the mode of action of these bioactives. Finally, most genes are directly related to insulin or glucose metabolism, but there is a great need to study genes involved in other important metabolic processes, such as inflammation, apoptosis, and oxidative stress, which may be linked to the prevention and treatment of the disease in an indirect way, it is also important to recognize that the genetic variants of each gene may respond differently to the compounds. It is noteworthy that most genes are also associated with other chronic diseases, which could encourage further studies on the subject.

## Author Contributions

KF, JG, LB-S, and IG contributed to the conception and design and drafted and critically revised the manuscript. All authors gave final approval and contributed to the article and approved the submitted version.

### Conflict of Interest

The authors declare that the research was conducted in the absence of any commercial or financial relationships that could be construed as a potential conflict of interest.
